# Effect of perioperative seizures on mortality and recurrence in patients with brain metastases

**DOI:** 10.3389/fonc.2022.1048304

**Published:** 2022-11-14

**Authors:** Yilong Zheng, Yuxiu Yang, Min Hui Ng, Adrienne Yu Hsiang Chew, Chun Peng Goh, Christopher Yuan Kit Chua, Rahul Rathakrishnan, Yvonne Ang, Andrea Li Ann Wong, Balamurugan Vellayappan, Kejia Teo, Vincent Diong Weng Nga, Tseng Tsai Yeo, Mervyn Jun Rui Lim

**Affiliations:** ^1^ Yong Loo Lin School of Medicine, National University of Singapore, Singapore, Singapore; ^2^ Division of Neurosurgery, National University Health System, Singapore, Singapore; ^3^ Division of Neurology, National University Health System, Singapore, Singapore; ^4^ Department of Hemotology-Oncology, National University Health System, Singapore, Singapore; ^5^ Department of Radiation Oncology, National University Health System, Singapore, Singapore

**Keywords:** epilepsy, brain cancer, brain tumor, survival, resection

## Abstract

**Objective:**

To identify the independent risk factors for 30-day perioperative seizures, as well as to evaluate the effect of perioperative seizures on overall mortality and tumor recurrence among patients who underwent surgical resection of brain metastases.

**Methods:**

Patients who underwent surgical resection of brain metastases at our institution between 2011 and 2019 were included. 30-day perioperative seizures were defined as the presence of any preoperative or postoperative seizures diagnosed by a neurosurgeon or neurologist within 30 days of metastases resection. Independent risk factors for 30-day perioperative seizures were evaluated using multivariate logistic regression models. Kaplan-Meier plots and Cox regression models were constructed to evaluate the effects of 30-day perioperative seizures on overall mortality and tumor recurrence. Subgroup analyses were conducted for 30-day preoperative and 30-day postoperative seizures.

**Results:**

A total of 158 patients were included in the analysis. The mean (SD) age was 59.3 (12.0) years, and 20 (12.7%) patients had 30-day perioperative seizures. The presence of 30-day preoperative seizures (OR=41.4; 95% CI=4.76, 924; p=0.002) was an independent risk factor for 30-day postoperative seizures. Multivariate Cox regression revealed that any 30-day perioperative seizure (HR=3.25; 95% CI=1.60, 6.62; p=0.001) was independently and significantly associated with overall mortality but not tumor recurrence (HR=1.95; 95% CI=0.78, 4.91; p=0.154).

**Conclusions:**

Among patients with resected brain metastases, the presence of any 30-day perioperative seizure was independently associated with overall mortality. This suggests that 30-day perioperative seizures may be a prognostic marker of poor outcome. Further research evaluating this association as well as the effect of perioperative antiepileptic drugs in patients with resected brain metastases may be warranted.

## Introduction

Brain metastases are the most common tumors of the central nervous system, with an incidence three to four-fold higher than primary brain tumors (1–3). Among patients with brain metastases, the reported incidence of preoperative seizures ranged from 16.8 to 21.4% (2, 4, 5), while the reported incidence of postoperative seizures ranged from 7.8 to 15.4% (4, 6). The incidence of seizures varies across different primary tumor pathologies (7). In their cohort of patients with brain metastases, Oberndorfer et al. reported that seizures were the commonest among patients with metastatic melanoma (67%), lung metastases (29%), and gastrointestinal metastases (21%) (8).

Risk factors for preoperative seizures among patients with brain metastases include preoperative headache, cognitive deficits, multiple intracranial tumors, bone infiltration by tumor, and presence of metastases in the temporal, occipital, and/or frontal lobe (2, 5), while risk factors for postoperative seizures include presence of preoperative seizures, younger age, absence of cerebellar metastases, smaller tumor size, absence of preoperative headache, presence of parietal metastases, and checkpoint inhibitor use (2, 4, 5).

Data on the effect of perioperative seizures on mortality and tumor recurrence among patients with brain metastases is limited. There was only one study which examined the effect of preoperative seizures on mortality, and the study found no statistically significant association between preoperative seizures and mortality (5). To our knowledge, there are no studies which examined the association between perioperative seizures and tumor recurrence.

Given that the presence of perioperative seizures has been shown to affect prognosis among patients with glioblastoma (9), we hypothesized that the presence of perioperative seizures would affect prognosis among patients with brain metastases as well. Knowledge of the effect of perioperative seizures on outcome is important as it may have implications on the role of surgery and antiepileptic drugs for patients with perioperative seizures.

Therefore, we aimed to investigate the effect of 30-day perioperative seizures on overall mortality and tumor recurrence. We also aimed to identify the independent risk factors for 30-day perioperative seizures among patients with resected brain metastases.

## Methods

### Study design

A retrospective study of patients who underwent surgical resection of brain metastases at the National University Hospital, Singapore between 2011 and 2019 was conducted. Eligible patients were identified from the institutional electronic operative records system. Patients who (1) were 18 years old or above on the day of surgical resection and (2) had histologically confirmed brain metastasis were included. Our study was approved by the institutional review board prior to commencement (National Healthcare Group Domain Specific Review Board; Reference Number 2020/00358). A waiver of informed consent from patients was granted for this study.

### Data collection

Data were collected from the patients’ electronic medical records. The exposures for our study were 30-day perioperative seizures (seizure within 30 days before and/or after surgical resection), 30-day preoperative seizures (seizure within 30 days before surgical resection), and 30-day postoperative seizures (seizure within 30 days after surgical resection). Only seizures that were diagnosed by a neurosurgeon or neurologist were classified as a seizure. Clinical presentations which resembled a seizure but were not considered a seizure by a neurosurgeon or neurologist were not classified as a seizure. The outcomes analyzed in our study were overall mortality and tumor recurrence. Overall mortality was defined as death due to any reason documented in the electronic medical records, and tumor recurrence was identified on post-operative follow-up magnetic resonance imaging (MRI) of the brain reports.

Potential confounders that were collected included age, sex, ethnicity (defined as Chinese, Indian, Malay, or Others), perioperative antiepileptic drug (AED) use, history of epilepsy, site of primary tumor (categorized to the five commonest sites and others), presence of extracranial metastasis preoperatively, number of brain metastases, locations of the brain metastases, volume of the largest resected tumor (computed using AP x ML x CC/2, where AP, ML, and CC denote the greatest anterior-posterior, medial-lateral, and cranio-caudal diameters respectively), presence of hydrocephalus, and the extent of midline shift on preoperative MRI of the brain. Data collected on the surgical resection of the brain metastases included the number of tumors resected, presence of unresected tumors postoperatively, extent of resection (defined as gross total or subtotal resection), and whether there was residual tumor reported on postoperative MRI.

### Statistical analysis

Data for continuous variables were presented as mean and standard deviation, while data for categorical variables were presented as count numbers and percentages. For univariate analyses, we used Pearson’s chi-squared test for categorical variables and the student’s t-test for continuous variables. The Fisher’s exact test was used for variables with a sample size of lesser than or equal to 5 under any category. A p-value of less than 0.050 was considered statistically significant. To identify the independent risk factors for 30-day preoperative and 30-day postoperative seizures, multivariate logistic regression models were constructed. Variables that had a p-value of lesser than 0.100 on univariate analysis were included in the models. Variables with a p-value of lesser than 0.050 were considered statistically significant.

Time-to-event analysis was performed using the Kaplan-Meier method. Time-to-overall mortality was defined as the duration between the date of surgical resection and the date of death for patients who have passed away. Time-to-recurrence was defined as the duration between the date of surgical resection and the date of the first recurrence for patients who had tumor recurrence on follow up MRI. Hypothesis testing was conducted using the log-rank test, and a p-value of lesser than 0.05 was considered statistically significant. Cox-proportional hazard models were computed to evaluate the effects of 30-day perioperative seizures on overall mortality and tumor recurrence. Subgroup analyses were also conducted for 30-day preoperative seizures and 30-day postoperative seizures. Known confounders based on existing literature were adjusted for in three separate models (2, 4, 5). In the first model, we adjusted for baseline demographics, including age, gender, and ethnicity. In the second model, we adjusted for variables in the first model, as well as tumor characteristics, including tumor location in the temporal lobe, parietal lobe, or cerebellum, and volume of the largest resected tumor. In the third model, we adjusted for variables in the second model, as well as history of epilepsy and perioperative AED use. All data analyses were conducted on R Studio Version 1.2.5042 (10).

## Results

### Baseline characteristics of the study population

The baseline characteristics of the study population were reported in **Table 1**. A total of 158 patients were included in the analysis. The mean (SD) age was 59.3 (12.0) years, and 84 (53.2%) patients were female. A total of 20 (12.7%) patients had a 30-day perioperative seizure. 14 (8.9%) patients had a 30-day preoperative seizure, while 10 (6.3%) patients had a 30-day postoperative seizure.

**Table 1:** Baseline characteristics of patients with brain metastases with and without any 30-day perioperative seizure.

Variable	No perioperative seizure (n=138, 87.3%)	Perioperative seizure (n=20, 12.7%)	Total (n=158)	p-value
Age; mean (SD)	60 (11.6)	54.6 (13.9)	59.3 (12.0)	0.058
Female; *n* (%)	77 (55.8)	7 (35.0)	84 (53.2)	0.133
Ethnicity; *n* (%)				0.572
Chinese	94 (68.1)	13 (65.0)	107 (67.7)	
Malay	19 (13.8)	5 (25.0)	24 (15.2)	
Indian	7 (5.1)	0 (0.0)	7 (4.4)	
Others	18 (13.0)	2 (10.0)	20 (12.7)	
AED administered preoperatively; *n* (%)	53 (38.4)	15 (75.0)	68 (43.0)	**0.004**
AED administered immediately postoperatively; *n* (%)^1^	138 (100.0)	15 (75.0)	153 (96.8)	**<0.001**
History of epilepsy; *n* (%)	0 (0.0)	2 (10.0)	2 (1.3)	**0.015**
Number of brain metastases on preoperative MRI; mean (SD)	2.7 (4.5)	2.4 (1.9)	2.6 (4.3)	0.763
Site of primary tumor; *n* (%)				0.136
Lung	55 (39.9)	8 (40.0)	63 (39.9)	
Breast	36 (26.1)	4 (20.0)	40 (25.3)	
Colorectum	18 (13.0)	0 (0.0)	18 (11.4)	
Kidney	4 (2.9)	2 (10.0)	6 (3.8)	
Skin	5 (3.6)	2 (10.0)	7 (4.4)	
Others	20 (14.5)	4 (20.0)	24 (15.2)	
Presence of extracranial metastasis preoperatively; *n* (%)	85 (61.6)	15 (75.0)	100 (63.3)	0.361
Presence of unresected tumors postoperatively; *n* (%)	57 (41.6)	9 (45.0)	66 (42.0)	0.964
Presence of tumor(s) by location; *n* (%)				
Temporal	30 (21.7)	3 (15.0)	33 (20.9)	0.768
Frontal	50 (36.2)	8 (40.0)	58 (36.7)	0.937
Occipital	36 (26.1)	5 (25.0)	41 (25.9)	1.000
Parietal	43 (31.2)	10 (50.0)	53 (33.5)	0.157
Cerebellum	65 (47.1)	6 (30.0)	71 (44.9)	0.232
Deep brain	9 (6.5)	2 (10.0)	11 (7.0)	0.919
Volume of largest resected tumor preoperatively (cm^3^)^2^; mean (SD)	22.0 (35.2)	19.6 (29.6)	21.7 (34.5)	0.775
Preoperative hydrocephalus; *n* (%)	28 (20.3)	5 (25.0)	33 (20.9)	0.849
Preoperative midline shift (mm); mean (SD)	2.8 (4.4)	3.0 (4.9)	2.9 (4.4)	0.905
Gross total resection; *n* (%)	107 (77.5)	15 (75.0)	122 (77.2)	1.000
Presence of residual tumor postoperatively; *n* (%)	36 (26.1)	5 (25.0)	41 (25.9)	1.000

^1^This excludes patients who only received AED(s) to abort a postoperative seizure.

^2^Volume of tumor was estimated using the formula: Volume = (AP x ML x CC)/2

SD, standard deviation; AED, antiepileptic drug; MRI, magnetic resonance imaging; AP, anterior-posterior diameter; ML, medial-lateral diameter; CC, craniocaudal diameter.

Bold values means Statistically significant association.

### Risk factors for perioperative seizures

On univariate analysis, there was a statistically significant association between the presence of 30-day preoperative seizure and younger age (p=0.006), administration of preoperative AEDs (p<0.001), history of epilepsy (p=0.001), and absence of cerebellar metastases (p=0.022). There was a statistically significant association between the presence of a 30-day postoperative seizure and not administering AEDs immediately after surgery (p<0.001), presence of 30-day preoperative seizures (p=0.006), and smaller volume of the largest resected tumor (p=0.029. On multivariate analysis, there were no variables that had a statistically significant association with 30-day preoperative seizures. There was a statistically significant association between the presence of 30-day preoperative seizures (OR=41.4; 95% CI=4.76, 924; p=0.002) and the presence of 30-day postoperative seizures. There was no statistically significant association between administration of AEDs preoperatively and the presence of 30-day postoperative seizures (p=0.746).

### Association between 30-day perioperative seizures and overall mortality

The median (95% CI) overall survival of patients without any perioperative seizure was 12.5 (11.1, 16.7) months, while the median (95% CI) overall survival of patients with any 30-day perioperative seizure was 3.9 (2.9, 12.4), for 30-day preoperative seizures was 4.1 (3.8, NA), and for 30-day postoperative seizures was 3.0 (1.7, NA) months. The median (IQR) follow-up duration was 9.4 (17.9) months.

**Figure 1:**
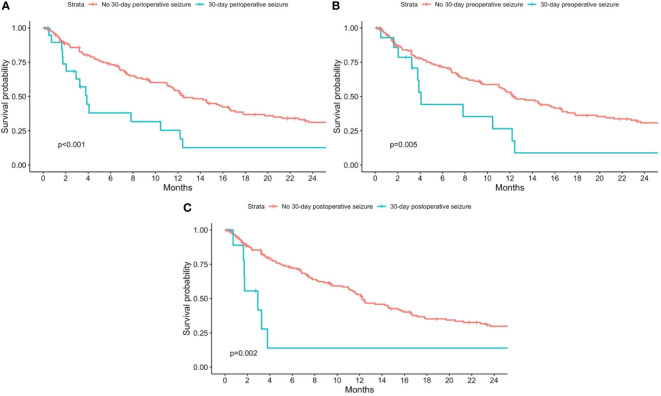
Kaplan-Meier curves for overall mortality stratified by patients with and without 30-day perioperative seizures. Patients with no 30-day perioperative seizures are shown in red, while patients with **(A)** any 30-day perioperative seizure; **(B)** 30-day preoperative seizure; and **(C)** 30-day postoperative seizure are shown in blue. P-values from the log-rank test are shown in each figure respectively.

On univariate analysis, there was a statistically significant association between overall mortality and the presence of any 30-day perioperative seizure (p<0.001), 30-day preoperative seizures (p=0.005), and 30-day postoperative seizures (p=0.002) (**Figures 1A–C**). On Cox-proportional hazards models, there was a statistically significant association between the presence of 30-day perioperative seizures and overall mortality (HR=3.25; 95% CI=1.60, 6.62; p=0.001), even after adjusting for baseline demographics, tumor characteristics, history of epilepsy, and perioperative AED use (**Table 2**). This association was also statistically significant for 30-day preoperative seizures (HR=3.06; 95% CI=1.52, 6.14; p=0.002). For 30-day postoperative seizures, the association was significant after adjusting for baseline demographics and tumor characteristics (HR=3.54; 95% CI=1.64, 7.64; p=0.001), but not after adjusting for history of epilepsy and preoperative AED use (HR=2.53; 95% CI=0.66, 9.67; p=0.176).

**Table 2:** Cox proportional hazards models showing the effect of 30-day perioperative seizures on overall mortality and recurrence of brain metastases.

Exposure	Mortality						Recurrence					
	Model 1		Model 2		Model 3		Model 1		Model 2		Model 3	
	HR (95% CI)	p-value	HR (95% CI)	p-value	HR (95% CI)	p-value	HR (95% CI)	p-value	HR (95% CI)	p-value	HR (95% CI)	p-value
Any 30-day perioperative seizure^1^	2.20 (1.29, 3.76)	**0.004**	3.26 (1.81, 5.88)	**<0.001**	3.25 (1.60, 6.62)	**0.001**	1.88 (0.82, 4.31)	0.138	1.93 (0.82, 4.55)	0.13	1.95 (0.78, 4.91)	0.154
30-day preoperative seizure^2^	1.97 (1.04, 3.73)	**0.036**	2.81 (1.42, 5.53)	**0.003**	3.06 (1.52, 6.14)	**0.002**	1.88 (0.77, 4.60)	0.165	1.93 (0.77, 4.84)	0.161	1.95 (0.78, 4.91)	0.154
30-day postoperative seizure^3^	2.91 (1.39, 6.08)	**0.005**	3.54 (1.64, 7.64)	**0.001**	2.53 (0.66, 9.67)	0.176	1.52 (0.35, 6.62)	0.577	1.54 (0.35, 6.72)	0.569	0.75 (0.08, 7.20)	0.804

Model 1: Adjusted for baseline demographics, including age, sex, and ethnicity.

Model 2: Adjusted for variables in Model 1 and characteristics of the tumor(s), including temporal location, parietal location, cerebellum location, and volume of the largest resected tumor on preoperative MRI.

Model 3: Adjusted for variables in Model 2, history of epilepsy, and perioperative AED use.

HR, hazard ratio; CI, confidence interval; AED, antiepileptic drug.

Bold values means Statistically significant association.

### Association between 30-day perioperative seizures and recurrence

**Figure 2:**
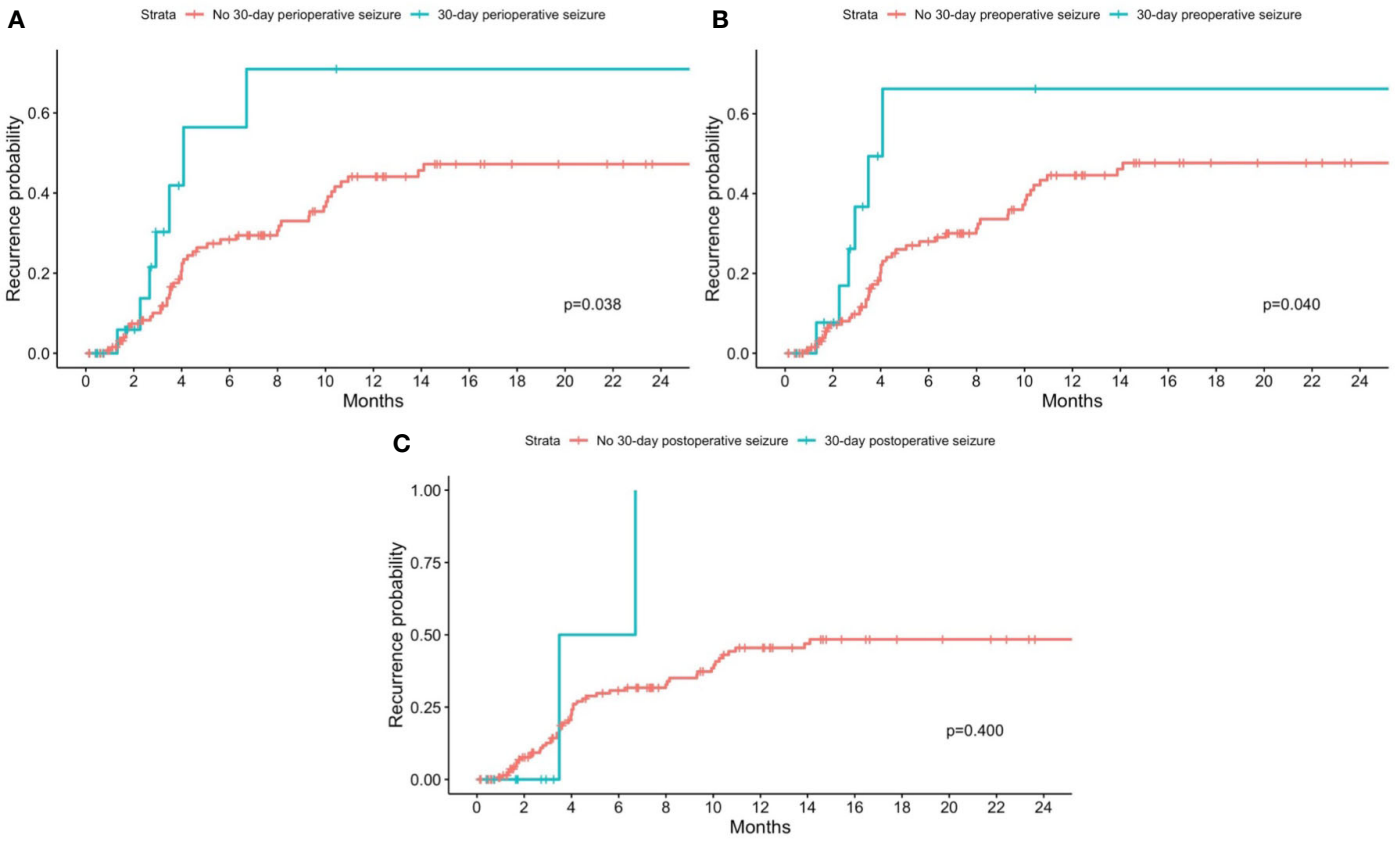
Kaplan-Meier curves for recurrence stratified by patients with and without 30-day perioperative seizures. Patients with no 30-day perioperative seizures are shown in red, while patients with **(A)** any 30-day perioperative seizure; **(B)** 30-day preoperative seizure; and **(C)** 30-day postoperative seizure are shown in blue. P-values from the log-rank test are shown in each figure respectively.

On univariate analysis, there was a statistically significant association between recurrence and the presence of any 30-day perioperative seizures (p=0.038) (**Figure 2A**) and 30-day preoperative seizures (p=0.040) (**Figure 2B**). There was however no statistically significant association between recurrence and presence of 30-day postoperative seizures (p=0.400) (**Figure 2C**). On cox-proportional hazards models, there was no statistically significant association between tumor recurrence and presence of any 30-day perioperative seizure, 30-day preoperative seizures, and 30-day postoperative seizures (**Table 2**).

## Discussion

In our cohort of patients who underwent surgical resection of brain metastases, the incidence of 30-day preoperative and 30-day postoperative seizures were 8.9% and 6.3% respectively. These rates are lower than rates reported in the literature (4, 6, 11–13). The reported incidence of preoperative seizures among patients with brain metastases ranged from 16.8 to 21.4% (2, 4, 5), while the reported incidence of postoperative seizures ranged from 7.8 to 15.4% (4, 6). A possible explanation for the relatively lower incidence of perioperative seizures could be that most patients in our cohort (96.8%) were prescribed prophylactic AEDs perioperatively, contrary to the practices of other institutions (4, 6, 14).

Our analyses did not reveal any independent risk factors for 30-day preoperative seizures but revealed the presence of 30-day preoperative seizures to be an independent risk factor for 30-day postoperative seizures, corroborating the findings of Wu et al. (2). Strikingly, administration of AEDs preoperatively was not significantly associated with the presence of 30-day postoperative seizures. 24

We also found that the presence of perioperative seizures was associated with a higher risk of recurrence and mortality. This could be because seizures are a result of increased neuronal activity, and neuronal activity has been shown to promote the growth of brain metastases (15).

However, the only study that examined the association between perioperative seizures and outcomes among patients with brain metastases reported no statistically significant association between preoperative seizures and mortality, though the effect of postoperative seizures on outcomes was not examined (5). Given the inconsistency between our and Garcia et al.’s findings (5), further studies evaluating the association between perioperative seizures and outcomes among patients with brain metastases are warranted, so that the role of perioperative seizures on outcomes can be explicated.

The effect of perioperative seizures on outcomes among patients with glioblastoma, on the other hand, is relatively well-studied. A meta-analysis of observational studies reported that preoperative seizures were associated with improved survival among patients with glioblastoma (9). Possible reasons for this correlation include the fact that seizures at presentation were associated with favorable genotypes (16, 17), because tumors that presented as seizures tended to be smaller, allowing for greater extent of resection and therefore improved survival (9), and also because seizures are generally more concerning to patients as compared to other milder symptoms of glioblastoma such as headache, leading to earlier diagnosis and therefore treatment (18). In addition, among patients with glioblastoma, early postoperative seizures were associated with poorer survival (19), and this association was hypothesized to be due to there being more extensive intracranial disease among patients with early postoperative seizures compared to those without early postoperative seizures (19).

The association between postoperative seizures and outcomes is consistent between brain metastases and glioblastoma – patients with postoperative seizures have a poorer prognosis. On the other hand, the association between preoperative seizures and outcomes is conflicting between patients with brain metastases and glioblastoma. Among patients with brain metastases, presence of preoperative seizures was either associated with a poorer outcome (as we have found) or was not associated with outcome (5). However, among patients with glioblastoma, the presence of preoperative seizures was associated with a better outcome (9). Garcia et al. attributed this to the fact that among patients with brain metastases, any survival benefit conferred by preoperative seizures was outweighed by the higher intracranial or systemic tumor burden (5).

To our knowledge, this is one of the first few studies evaluating the association between perioperative seizures and overall mortality and recurrence among patients with resected brain metastases. Our study was limited by the small sample size of our cohort, which may limit the statistical power of our analysis. However, despite the small sample size, we were able to show a statistically significant association between 30-day perioperative seizures and overall mortality in our cohort. However, as our study was a single-center study on patients with resected brain metastases, our conclusions may not be generalizable to other cohorts or patients with non-surgically treated brain metastases. Also, the 30-day timeframe for the occurrence of seizures may have been overly restrictive in retrospect as based on our argument that seizures promote the growth of tumor and therefore recurrence, the timepoint in which the seizures occurred should not matter. Future studies should evaluate whether the presence of seizures at any point in time affect outcomes among patients with brain metastases. Lastly, as our study was a retrospective study, we included only seizures that were documented in the clinical notes and there may be the potential for misclassification of the exposure if there were unwitnessed or unreported seizures. However, as seizures are a clinically significant event, we hypothesize that the rate of unwitnessed and/or unreported seizures in this cohort of resected brain metastases were likely to be low, and any misclassification of the exposures were likely to be non-differential.

## Conclusions

Among patients with resected brain metastases, the presence of any 30-day perioperative seizure was independently associated with overall mortality. This suggests that 30-day perioperative seizures may be a prognostic marker of poor outcome. Further research evaluating this association as well as the effect of perioperative AEDs in patients with resected brain metastases may be warranted.

## Data availability statement

The datasets presented in this article are not readily available because of local regulations. Requests to access the datasets should be directed to mervynlim@u.nus.edu.

## Ethics statement

The studies involving human participants were reviewed and approved by National Healthcare Group Domain Specific Review Board. Written informed consent for participation was not required for this study in accordance with the national legislation and the institutional requirements.

## Author contributions

All authors listed have made a substantial, direct, and intellectual contribution to the work and approved it for publication.

## Conflict of interest

The authors declare that the research was conducted in the absence of any commercial or financial relationships that could be construed as a potential conflict of interest.

## Publisher’s note

All claims expressed in this article are solely those of the authors and do not necessarily represent those of their affiliated organizations, or those of the publisher, the editors and the reviewers. Any product that may be evaluated in this article, or claim that may be made by its manufacturer, is not guaranteed or endorsed by the publisher.
